# Effects of Different Mold Materials and Coolant Media on the Cooling Performance of Epoxy-Based Injection Molds

**DOI:** 10.3390/polym14020280

**Published:** 2022-01-11

**Authors:** Chil-Chyuan Kuo, Jing-Yan Xu, Yi-Jun Zhu, Chong-Hao Lee

**Affiliations:** 1 Department of Mechanical Engineering, Ming Chi University of Technology, New Taipei City 24301, Taiwan; U09217119@mail.mcut.edu.tw (J.-Y.X.); M09118003@mail.mcut.edu.tw (Y.-J.Z.); U09117014@mail.mcut.edu.tw (C.-H.L.); 2 Research Center for Intelligent Medical Devices, Ming Chi University of Technology, No. 84, Gungjuan Road, New Taipei City 24301, Taiwan

**Keywords:** conformal cooling channel, rapid tooling technology, mold material, cooling medium

## Abstract

Metal additive manufacturing techniques are frequently applied to the manufacturing of injection molds with a conformal cooling channel (CCC) in order to shorten the cooling time in the injection molding process. Reducing the cooling time in the cooling stage is essential to reducing the energy consumption in mass production. However, the distinct disadvantages include higher manufacturing costs and longer processing time in the fabrication of injection mold with CCC. Rapid tooling technology (RTT) is a widely utilized technology to shorten mold development time in the mold industry. In principle, the cooling time of injection molded products is affected by both injection mold material and coolant medium. However, little work has been carried out to investigate the effects of different mold materials and coolant media on the cooling performance of epoxy-based injection molds quantitatively. In this study, the effects of four different coolant media on the cooling performance of ten sets of injection molds fabricated with different mixtures were investigated experimentally. It was found that cooling water with ultrafine bubble is the best cooling medium based on the cooling efficiency of the injection molded parts (since the cooling efficiency is increased further by about 12.4% compared to the conventional cooling water). Mold material has a greater influence on the cooling efficiency than the cooling medium, since cooling time range of different mold materials is 99 s while the cooling time range for different cooling media is 92 s. Based on the total production cost of injection mold and cooling efficiency, the epoxy resin filled with 41 vol.% aluminum powder is the optimal formula for making an injection mold since saving in the total production cost about 24% is obtained compared to injection mold made with commercially available materials.

## 1. Introduction

Wax injection molding is a frequently applied manufacturing technique for the production of investment casting wax patterns due to time efficiency. The cooling time of the wax patterns constitutes a large portion in cycle time. Nowadays, the conformal cooling channel (CCC) was incorporated in the wax injection mold to improve the cooling efficiency of the wax patterns in terms of uniform cooling during the cooling stage [1]. Low-pressure wax injection molding is one of the most widely used approaches for producing wax patterns, which can be used for investment casting [2] to manufacture metal components with intricate shapes [3]. Kitayama et al. [4] optimized the process parameters of plastic injection molding to reduce warpage of the plastics products using CCC. This work confirmed that the CCC provides the reduction on the warpage of the injection molded part. Rajaguru et al. [5] employed commercially available aluminum (Al)-filled epoxy resin to make a cavity insert for low-volume production of plastic parts. The tool life is acceptable under general process parameters. Lim et al. [6] proposed a method for designing the cooling channel by means of the energy balance principle and arrangement method. It was found that both the tensile strengths and hardness of the roof side products were improved. Chen et al. [7] showed segmented finite element models to optimize the geometries of the cooling system. It was found that the computational time saves up to 92.6% compared with the entire model of the U-shape component. Additive manufacturing (AM) techniques are frequently used in various industries to create end-use parts or physical prototypes. Maji et al. [8] developed patient-specific craniofacial implant through the AM, rapid tooling (RT), and investment casting technologies. Gill and Kaplas [9] proposed rapid casting technologies to fabricate a tool. It was found that the proposed rapid casting technologies are effective for the production of cast technological prototypes in very short times avoiding any tooling phase and with dimensional tolerances that are completely consistent with metal casting processes. Nandi et al. [10] developed a new composite material to enhance the solidification efficiency in the RT process. It was found that the solidification time was minimized appreciably keeping the same advantages of RT process. Wang et al. [11] optimized the molds with spherical spiral conformal cooling system and product structure to reduce service stress of the injection molded parts. This work suggested that injection molding defects such as warpage and residual stress cannot be ignored, especially for the assembly edge. Mercado-Colmenero et al. [12] proposed a new method for the automatic design of CCC based on the discrete geometry of the plastic part. The CCC follows the profile of the mold core or cavity to perform a uniform cooling process for injection molding process. This work demonstrated that the proposed algorithm is independent of the computer-aided design (CAD) modeler used to create the part since it performs a recognition analysis of the part surfaces, being able to be implemented in any CAD system. Additionally, the design data can be utilized in later applications including the automated design of the injection mold.

The wax injection mold with highly complex-shaped CCC capable of reduction cooling times can be fabricated by the metal powder AM, such as laser fusing, direct metal laser sintering, electron beam melting, selective laser melting, selective laser sintering, diffusion bonding, or direct metal deposition. However, operating consumables of the metal powder AM systems are costly. Thus, those approaches are not suitable for developing a new product since higher initial cost of the capital equipment and maintenance. To overcome this obstacle, RT technology was developed to meet this requirement since it is a cost-effective method that builds tools without the need for complex conventional machining operations. Typically, the RT technology is divided into two categories, namely direct tooling and indirect tooling. Indirect tooling is widely used to manufacture an injection mold for batch production of a new product in the research and development stage using commercial Al-filled epoxy resin. According to practical experience, both mechanical and physical properties of the wax injection mold fabricated by Al-filled epoxy resin are limited due to intrinsic material properties. Tomori et al. [13] proposed a silicon carbide filled epoxy casting system intended for prototype molds in plastic injection molding applications. Results revealed that the flexural strength of the molded parts increased with silicon carbide concentration. Kovacs and Bercsey [14] investigated the influence of mold properties on the quality of injection molded parts. Results demonstrated that the rapid tool inserts are useful in the injection molding technology, although the warpage of the molded parts could be more significant. In addition, injection molding simulation programmers can analyze the cooling time of the molded part or minimize the warpage of the molded part using CCC. Khushairi et al. [15] proposed metal filled epoxy as an alternative material used in RT application for injection molding. Results showed that the compressive strength is increased from 76.8 to 93.2 MPa with 20% of brass fillers. In addition, metal fillers also enhance the thermal properties and density of Al-filled epoxy resin. Cost-effectiveness depends on the cooling time of the molded part greatly in the injection molding process. Metal additive manufacturing (MAM) [16] technology is applied to speed up manufacturing for the production of injection mold with CCC. However, the distinct disadvantage is expensive spending [17] and longtime taking [18] in the fabrication of injection mold with CCC. Under certain condition of less expenditure is possible to manufacture injection molds by RT technology with conformal cooling channel.

In general, the cooling time of injection molded products is affected by both injection mold material and coolant medium. However, little work has been conducted to investigate the effects of different mold materials and coolant media on the cooling performance of epoxy-based injection molds. The goal of this study is to investigate the cooling performance of epoxy-based injection molds fabricated from different mold materials using different coolant media. In this study, ten sets of injection molds were fabricated using commercially available Al-filled epoxy resin and epoxy resin added with three different particle sizes of stainless steel, Al, and copper (Cu) powders. Four different kinds of cooling media, including cooling water, compressed gas, cooling water with ultrafine bubble, and cold stream are used to study the cooling performance using a low-pressure wax injection molding.

## 2. Experimental Details

**Figure 1:**
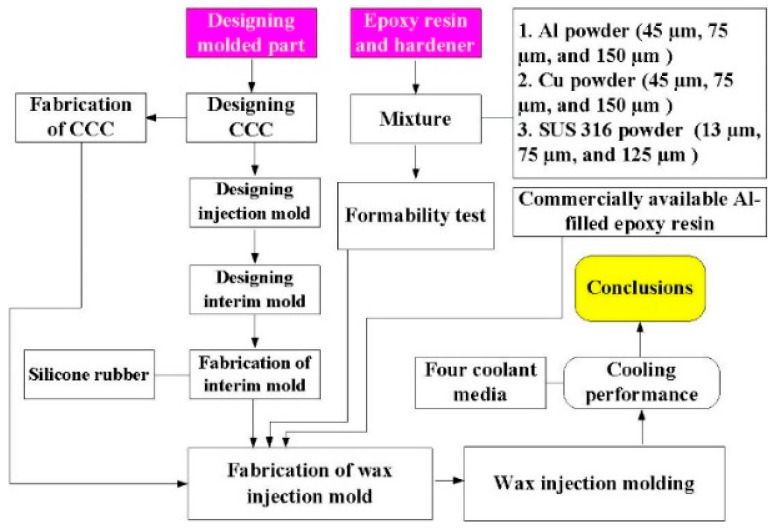
Research process of this study.

**Figure 2:**
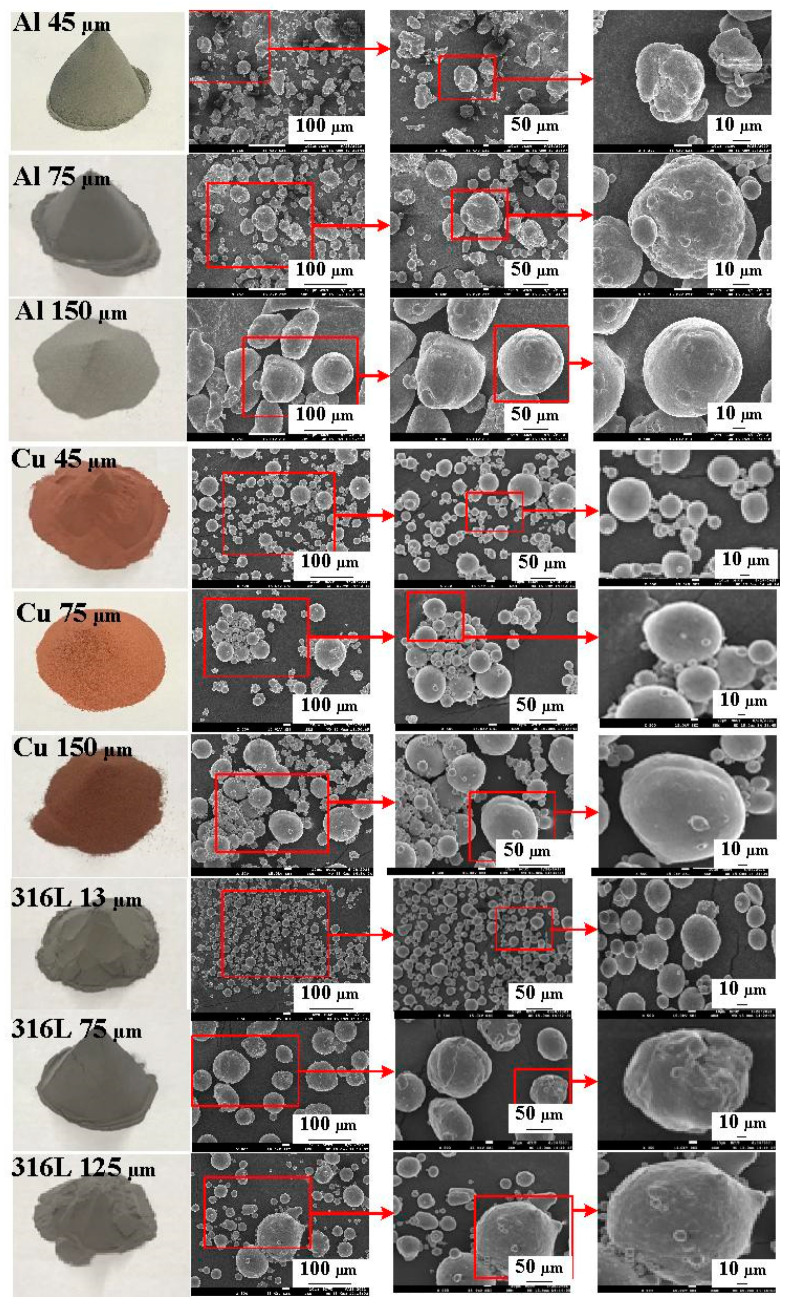
Three different kinds of fillers used in this study.

Figure 1 shows the research process of this study. The three different particle sizes of stainless steel (SUS) 316 powder, Al powder, and Cu powder were used in this study. Figure 2 shows the three different kinds of fillers with the purity of the fillers is approximately 96–99%. Average particle sizes of the three different kinds of fillers were examined by field-emission scanning electron microscopy (FE-SEM) (JEC3000-FC, JEOL Inc., Tokyo, Japan). The Al powder and Cu powder have three average particle sizes of 45 µm, 75 µm, and 150 µm, respectively. 316 stainless steel powder has three average particle sizes of 13 µm, 75 µm and 125 µm. The epoxy resin (EP-2N1, Ruixin Inc., Taipei, Taiwan) with the viscosity about 13,000 mPa-s was used as matrix material. The mixture is composed of epoxy resin, hardener, and filler. The curing agent and epoxy resin were mixed in a weight ratio of 1:2 first and then filler particle was added. The mixture was stirred manually about 10 min until the mixture is well blended. The mixture was then degassed by a vacuum pump (F-600, Feiling Inc., Taipei, Taiwan). Finally, the fabricated wax injection molds were post-cured in a thermal oven at 60 °C for obtaining desired strength to withstand injection and hold pressures. Note that the wax injection molds have very limited shrinkage after post-cured process. The thermal conduction of the wax injection mold fabricated by different mixtures was examined using a hot plate (YS-300S, Yong-Xin Inc., Taipei, Taiwan) with the heating temperature of 40. The chemical composition of the filler was examined using an energy-dispersive X-ray spectroscopy (EDS). The X-ray phase analysis was carried out using an X-ray diffractometer (XRD) (D8 ADVANCE, Bruker Inc., Taipei, Taiwan) to study the structures of the fabricated wax injection molds. To assess the benefits of the optimum material formulation, commercially available Al powder-filled epoxy resin (TE-375, Jasdi Chemicals, Inc., Taipei, Taiwan) was also employed to fabricate injection molds.

**Figure 3:**
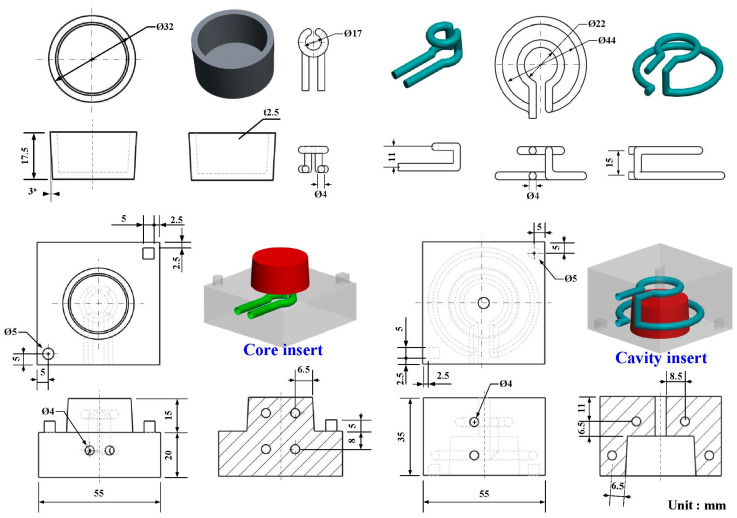
3D CAD model and dimensions of the CCC, core, and cavity inserts.

Figure 3 shows the 3D CAD model and dimensions of the CCC, core and cavity inserts. In this study, a pipe cover with the dimensions of 32 mm in outer diameter, 17.5 mm in height, and 2.5 mm in thickness was selected as the master pattern. The circular CCC was used in this study since it has the smallest pressure loss during the cooling stage [19]. According to the design guideline of the CCC, the distance between the wall of cooling channel to the mold surface, and the pitch distance between central lines of cooling channels are 4 mm, 7.5 mm, and 17 mm, respectively. Generally, there are various different types of CC (i.e., longitudinal, zigzag, spiral, parallel, scaffold, or Voronoi diagram). In this study, the spiral-shaped CCC was used in the wax injection mold. The core and cavity inserts have a length of 55 mm, a width of 55 mm, a height of 35 mm. Thanks to the distinct advantage of AM technique, the AM-based cooling channel was used to fabricate wax injection mold via fused filament fabrication using polyvinyl butyral (PVB) filament since the industrial alcohol solution is capable of dissolving the PVB plastic.

**Figure 4:**
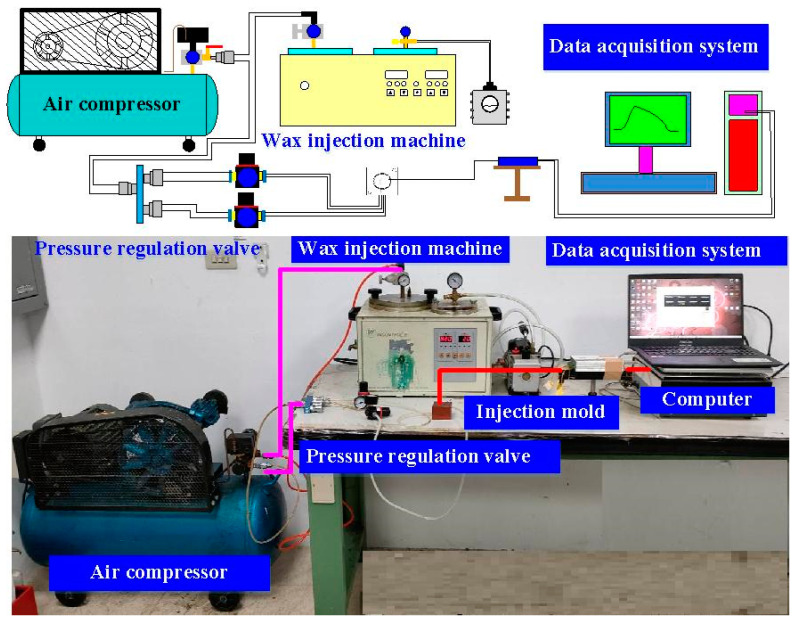
An in-house cooling time measurement system.

Cooling media is a substance, which is widely applied to regulate or reduce temperature of injection mold during the injection molding process. An ideal cooling medium involves low cost, low viscosity, high specific heat capacity, non-toxic, and chemically inert of the cooling system in the injection mold. Air is a common form of a cooling media, which uses forced circulation to reduce temperature of injection mold during the injection molding process. Thus, four different kinds of cooling media (i.e., compressed gas, cooling water, cooling water with ultrafine bubble, and cold stream) were applied to study the cooling efficiency of injection molds by low-pressure wax injection molding. Cold air comes from the vortex tube. To evaluate cooling performance of the injection molds, a simple and effective cooling time measurement system was designed and implemented. Figure 4 shows an in-house cooling time measurement system. This measurement system comprises a low-pressure wax injection molding machine (0660, W&W Inc., Taoyuan, Taiwan), three k-type thermocouples (C071009-079, Cheng Tay Inc., Taipei, Taiwan) with a measurement sensitivity of ±1 °C, a mold temperature controller (JCM-33A, Shinko Inc., Taipei, Taiwan), and a water reservoir with a thermo-electric cooler (TEC12706AJ, Caijia Inc., Taipei, Taiwan). The process parameters for low-pressure wax injection molding involve injection temperature of 95 °C, injection pressure of 0.06 MPa, and an ejection temperature of 30 °C. The temperature sensors were installed in the mold cavity. The other end of the temperature sensor was connected to a data acquisition system (MRD-8002L, IDEA System Inc., Taipei, Taiwan). With the help of the temperature sensors, the temperature histories of the injection molded wax patterns after low-pressure wax injection molding were recorded in time interval of 1 s for determining the cooling time of the injection molded wax patterns. The room temperature was kept at 27 °C for the entire study. The injection mold was held in horizontal position and the molten wax at 82 °C was injected into the mold cavity at 27 °C. The ejection temperature of the molded wax patterns was set at 30 °C via a series of test runs. The inlet coolant temperature was kept at room temperature.

## 3. Results and Discussion

**Figure 5:**
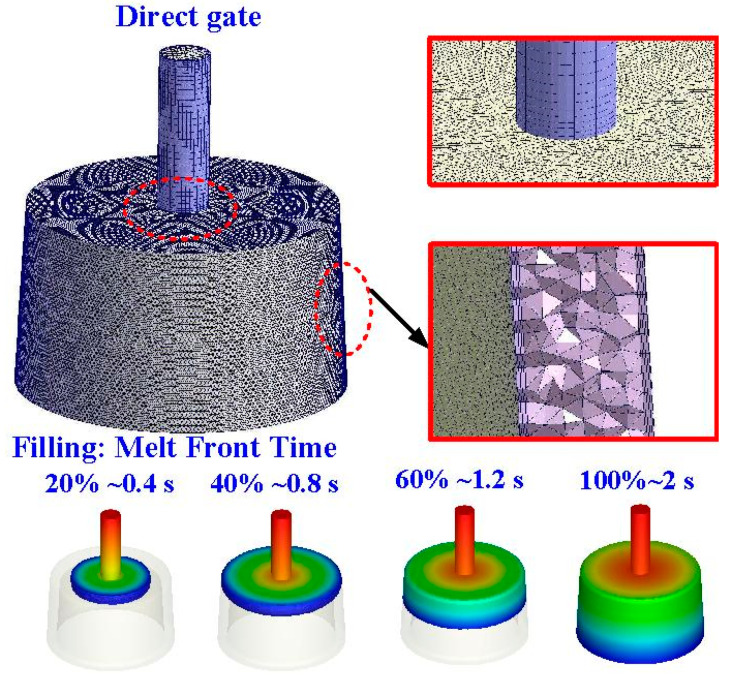
Mesh of the injection molded part and cooling channels.

To ensure the molding materials can fill the cavity completely and smoothly, a direct gate was employed in this study. Figure 5 shows the mesh of the injection molded part and cooling channels. The evolution of the flow front during the filling stage of wax injection molding is also illustrated in this figure. It shows that the filling time is approximately 2 s for the full filing of the molded part, showing the designed filling system is appropriate. In addition, no weld lines or air traps of the injection molded part were found at the end of filling.

**Figure 6:**
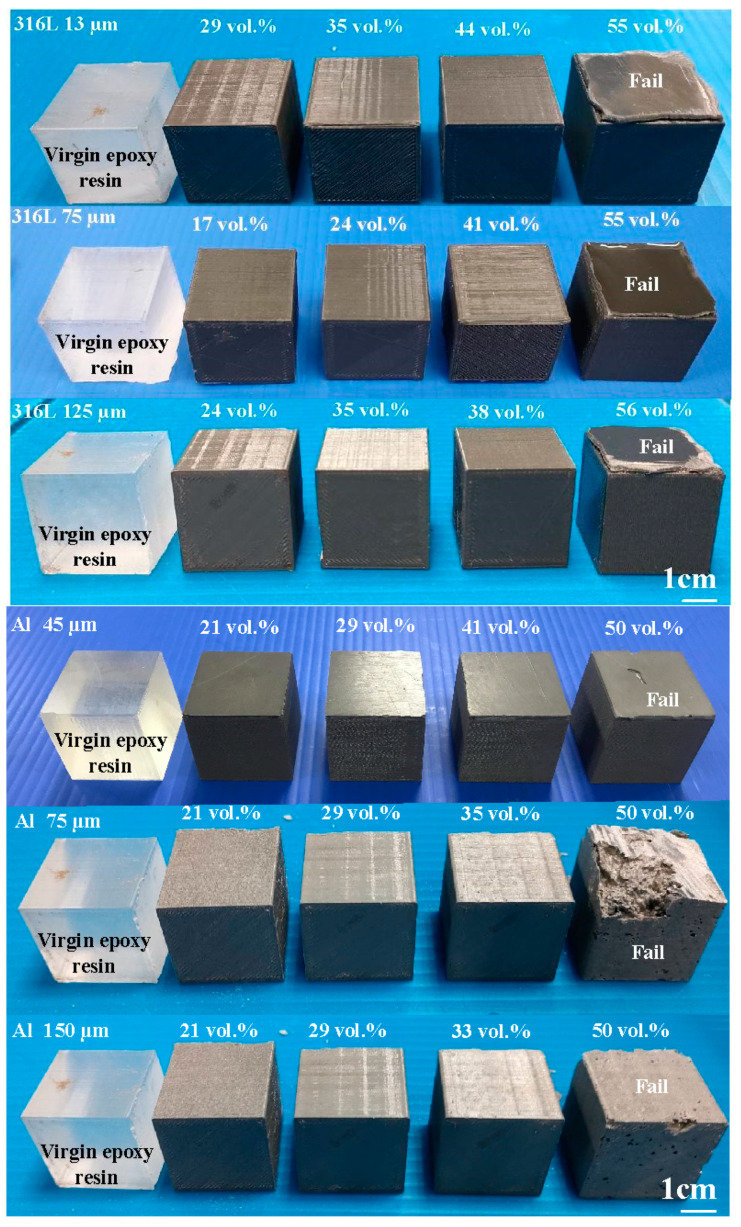

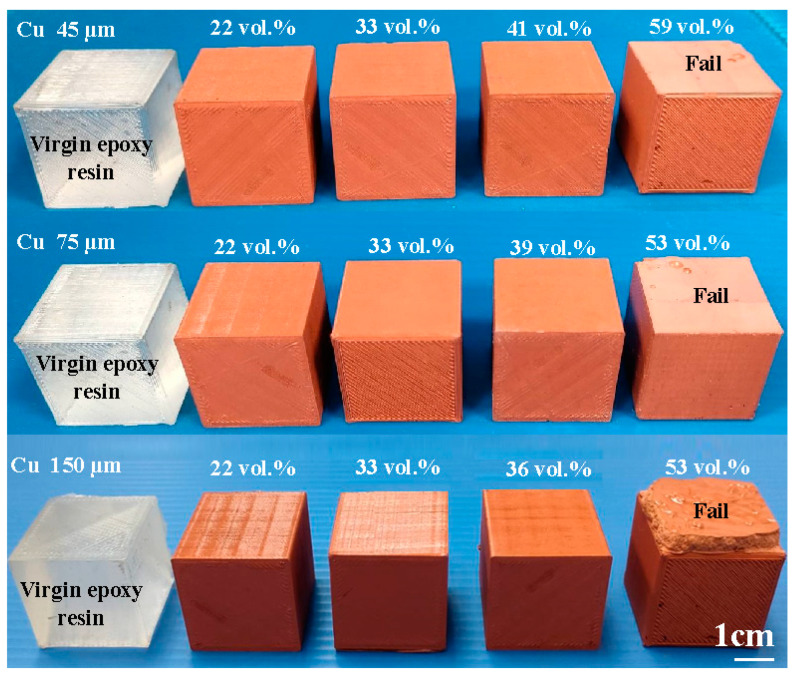
Formability results of the epoxy resin filled with three different powders with three different particle sizes.

To investigate the upper limit of the volume percentage (vol.%) of the filler particles that can be added to the matrix material of epoxy resin, the vol.% of these three powders was varied from 15 to 65 vol.%. Figure 6 shows the formability results of the epoxy resin filled with three different powders with three different particle sizes. Based on the formability and manufacturability of the specimens, the vol.% for 316 stainless steel powder with an average particle size of 13 µm, 75 µm, and 125 µm can be added to the epoxy resin are about 44 vol.%, 41 vol.% and 38 vol.%, respectively. The vol.% for Al powder with an average particle size of 45 µm, 75 µm, and 125 µm can be added to the epoxy resin are about 41 vol.%, 35 vol.% and 33 vol.%, respectively. The vol.% for Cu powder with an average particle size of 45 µm, 75 µm, and 125 µm can be added to the epoxy resin are about 41 vol.%, 39 vol.% and 36 vol.%, respectively.

**Figure 7:**
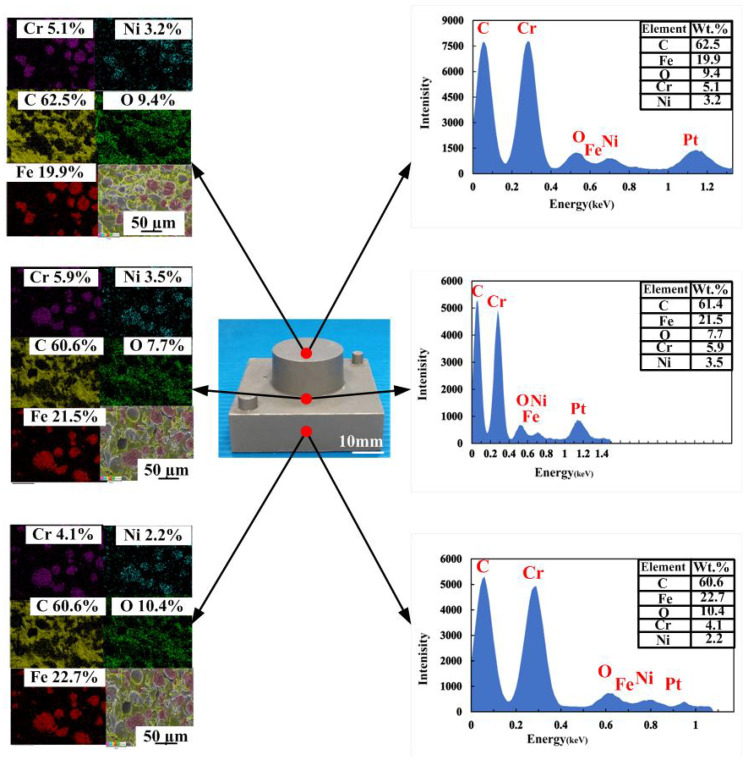
Precipitation analysis of the injection mold fabricated by epoxy resin filled with SUS 316 powder.

**Figure 8:**
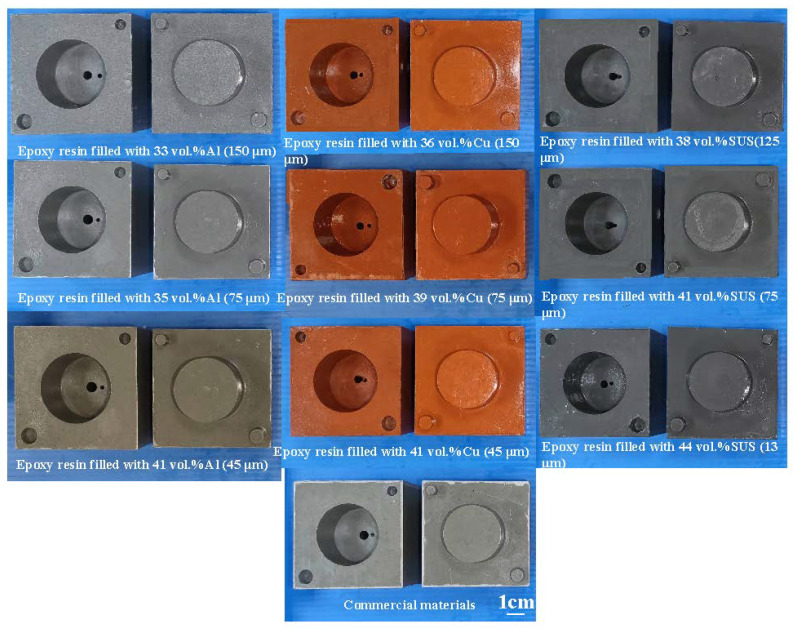
Ten pairs of injection molds for low-pressure wax injection molding.

**Figure 9:**
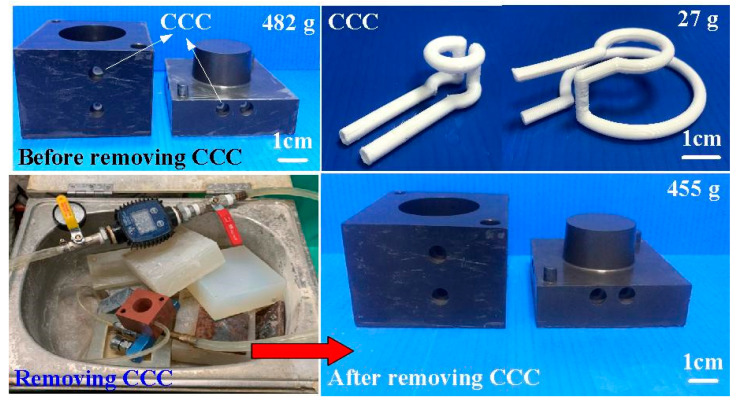
Results of before and after removing CCC.

To investigate the precipitation of fillers in the injection mold, EDS was carried out in this study. Figure 7 shows the precipitation analysis of the injection mold fabricated by epoxy resin filled with SUS 316 powder. As can be seen, the colors of the top, middle, and bottom of the injection mold are similar, showing that there is no any powder precipitation. This inference is supported by the EDS measurements of the wax injection mold. It was found that elements of Cr and Ni at the top, middle and bottom of the injection mold are similar. This means the filler of SUS 316 powder can be dispersed uniformly in the injection mold. Figure 8 shows the ten pairs of injection molds for low-pressure wax injection molding. Figure 9 shows the results of before and after removing CCC. The weight of the CCC is 27 g. The weight of the injection mold with CCC is 482 g. After removing the CCC, the weight of the injection mold without CCC is only 455 g. This result revealed that the CCC inside the injection mold was removed completely.

**Figure 10:**
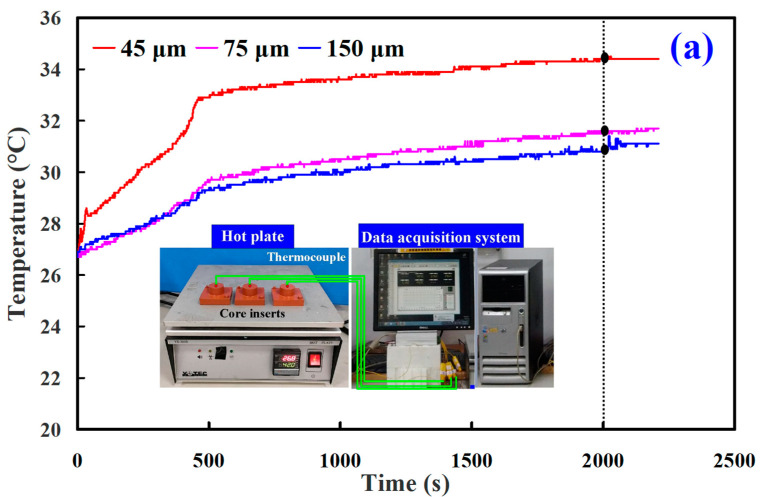

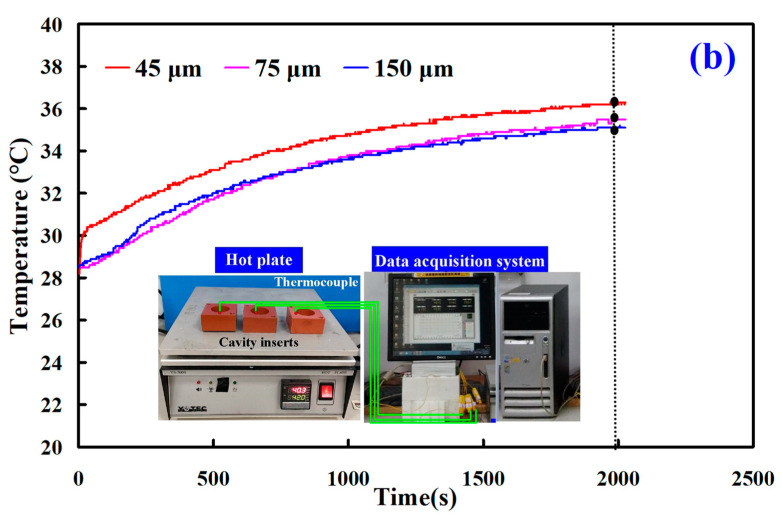
Results of the heat conduction experiment for (**a**) core and (**b**) cavity of the wax injection mold fabricated by epoxy resin filled with three different particle sizes of Cu powder.

**Figure 11:**
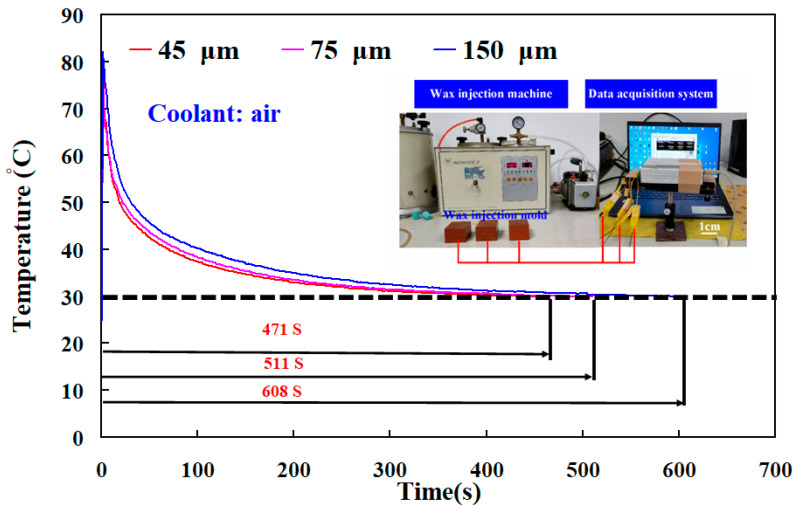
Temperature of wax pattern as a function of the cooling time.

**Figure 12:**
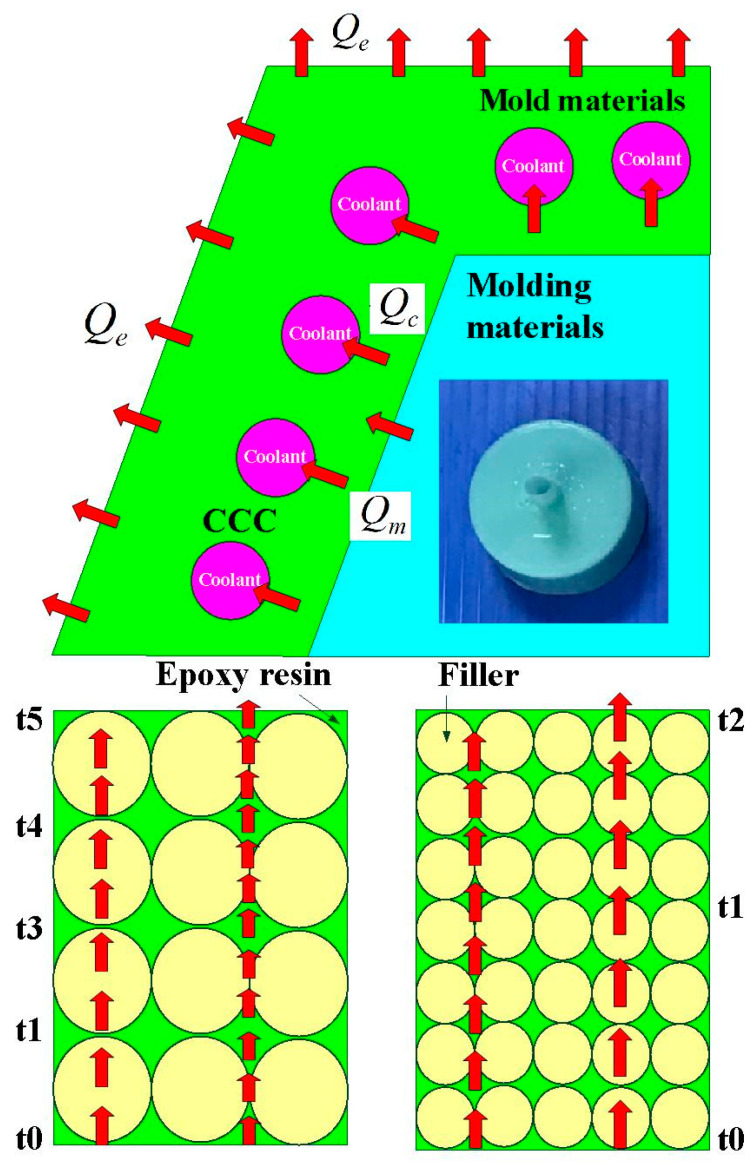
Schematic illustrations of heat conduction of the wax injection mold fabricated by epoxy resin filled with Cu powder with larger and smaller average particle sizes.

Figure 10 shows the results of the heat conduction experiment for core and cavity of the wax injection mold fabricated by epoxy resin filled with three different particle sizes of Cu powder. The results showed that the thermal conductivity of the wax injection mold fabricated by epoxy resin filled with average particle size of 45 µm Cu powder is the highest, followed by the wax injection mold fabricated by epoxy resin filled with average particle size 75 µm Cu powder. The wax injection mold fabricated by epoxy resin filled with average particle size 150 µm Cu powder has the lowest thermal conductivity. To further verify the heat transfer characteristics of the wax injection mold fabricated by epoxy resin filled with three different particle sizes of Cu powder, a series of experiment of low-pressure wax injection molding were carried out. Figure 11 shows the temperature of wax pattern as a function of the cooling time. The ejection temperature of the wax pattern was set as 30 ℃. Similarly, the cooling time of the wax pattern is affected by the particle size of Cu powder filled into the epoxy resin. The cooling time of the wax pattern fabricated by epoxy resin filled with 45 µm Cu powder is the shortest. The cooling times of the wax pattern duplicated by wax injection mold fabricated by epoxy resin filled with average particle size of 45 µm, 75 µm, and 150 µm Cu powder are around 471 s, 511 s, and 608 s, respectively. Thus, the wax injection mold fabricated by epoxy resin filled with Cu powder with a smaller average particle size has better heat conduction effect. The schematic illustrations of heat conduction of the wax injection mold fabricated by epoxy resin filled with Cu powder with larger and smaller average particle sizes are shown in Figure 12. It should be noted that the same phenomena were found when wax injection mold fabricated by epoxy resin filled with SUS or Al powder with a smaller average particle size.

**Figure 13:**
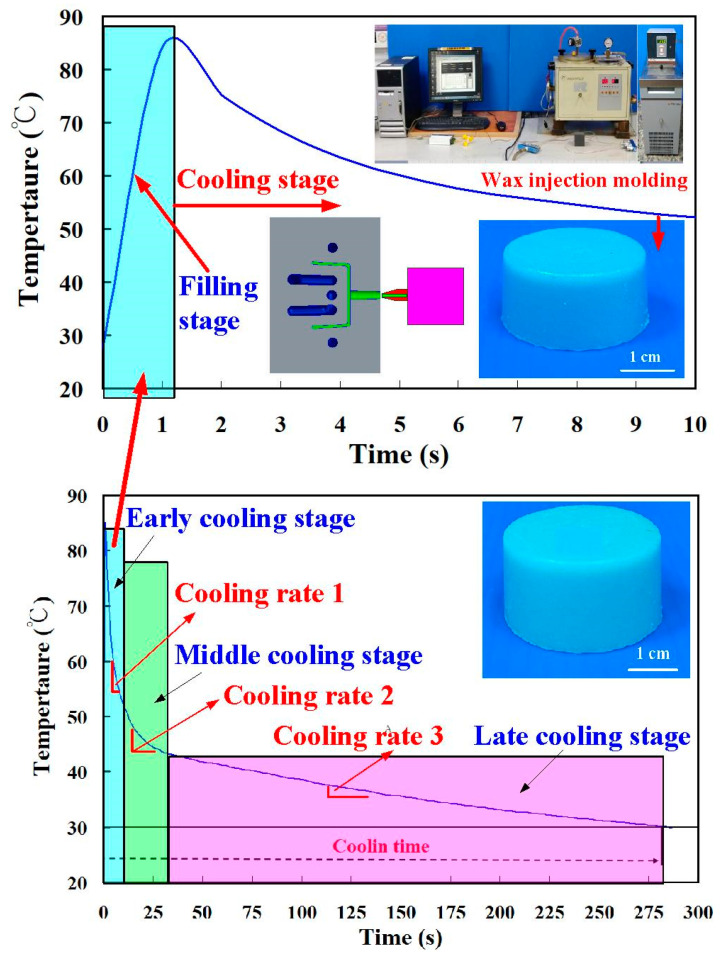
Typical temperature history of the injection molded part.

Figure 13 shows typical temperature history of the injection molded part after low-pressure wax injection molding. Temperature history involves four stages, including filling, early cooling, middle cooling, and late cooling stages. The cooling time of the wax patterns was defined the duration form the filling stage to the temperature of wax pattern reaching the ejection temperature of 30 ℃. The heat of the molten wax was removed by the coolant in the mold, which is recommended based on the solidification of the wax patterns. The cooling time (*t_c_*) of the injection molded parts can be calculated by the following equation [20,21,22]:

(1) $$t_{c}=\frac{s^{2}}{\pi^{2}\alpha }\ln\left[\frac{4}{\pi }\left(\frac{T_{m}-T_{w}}{T_{e}-T_{w}}\right)\right]$$

*α* is the thermal diffusivity;
*T_w_* is the mold cavity surface temperature;
*s* is the part thickness;
*T_m_* is the melt temperature;
*T_e_* is the average ejection temperature.

**Figure 14:**
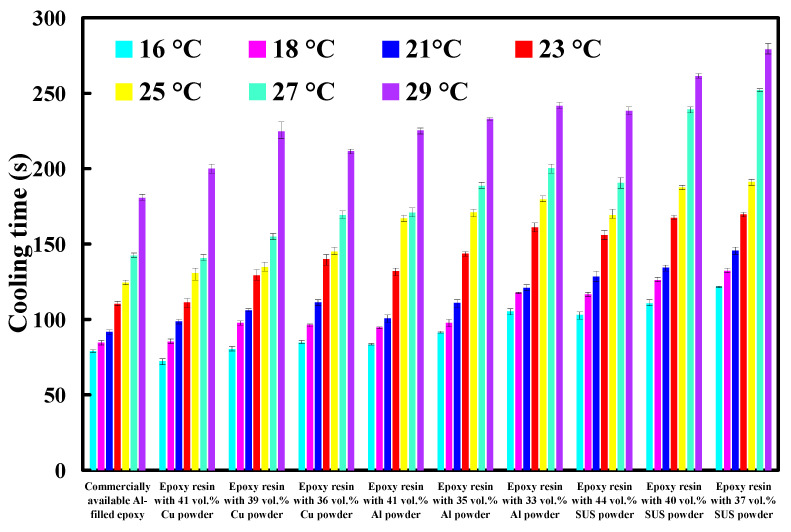
Cooling time of injection molded parts fabricated by ten sets of injection molds cooled with different cooling water temperatures.

**Figure 15:**
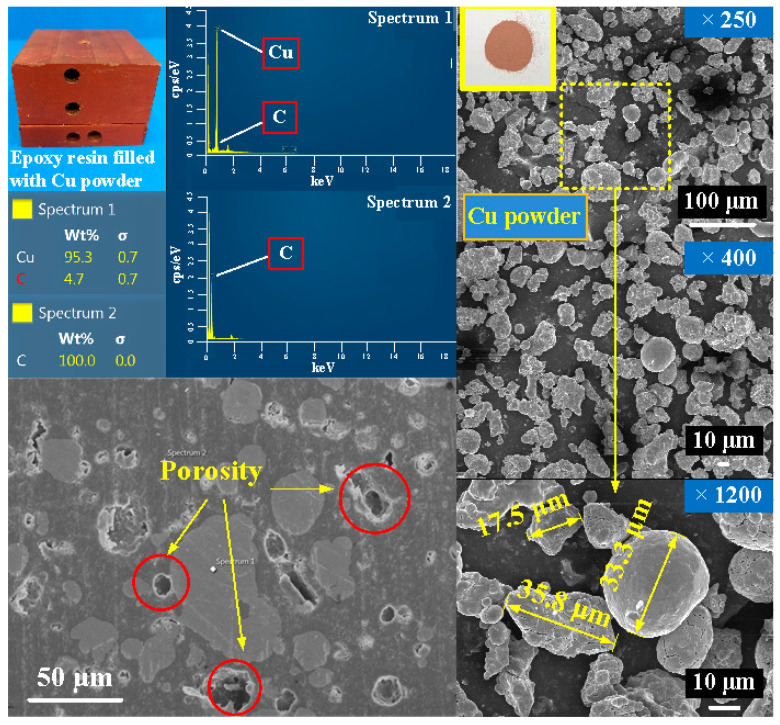
SEM micrograph of injection mold made with Cu-filled epoxy resin.

Figure 14 shows cooling time of injection molded parts fabricated by ten sets of injection molds cooled with different cooling water temperatures. Based on these results, four phenomena were found: (a) cooling time of the wax pattern is shorter when the cooling system uses a low temperature coolant; (b) cooling time of the wax pattern is not sensitive to injection mold with different thermal conductivity when the cooling system uses a higher temperature coolant; (c) cooling time of the wax pattern is sensitive to injection mold with different thermal conductivity when the cooling system uses a lower temperature coolant; and (d) cooling time of the wax pattern fabricated by injection mold made with Cu-filled epoxy resin is not shorter than that fabricated by injection mold made with commercially available Al-filled epoxy resin. Figure 15 shows the SEM micrograph of injection mold made with Cu-filled epoxy resin. The underlying reason is that the mixture is easier to generate porosity [23] when the Cu powder was added in the epoxy resin, which will affect the heat transfer effect of the fabricated injection mold.

**Figure 16:**
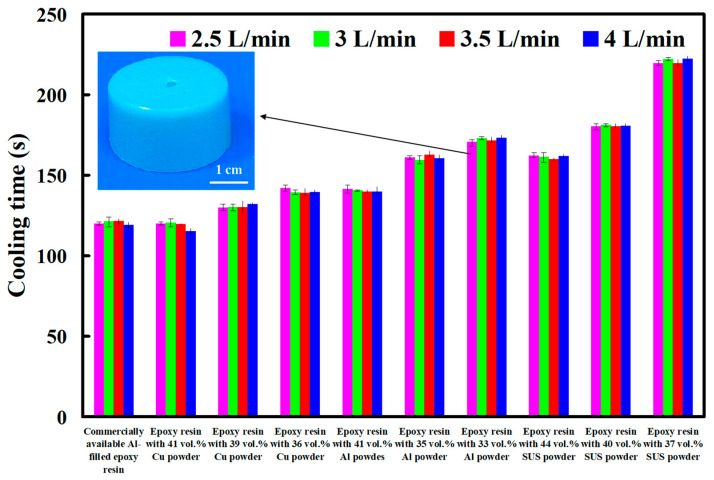
Cooling time of injection molded parts fabricated by ten sets of injection molds cooled with different cooling water flow rates.

Generally, the cooling water flow rate is an important issue in the cooling performance of injection mold with CCC. The turbulent flow [24] having a Reynolds number greater than 4000 provides three to five times as much heat transfer as laminar flow having a Reynolds number less than 2100 [25]. Figure 16 shows cooling time of injection molded parts fabricated by ten sets of injection molds cooled with different cooling water flow rates. The Reynolds number for four different coolant flow rates is about 4927, 5913, 6897, and 7883, respectively. As can be seen, the cooling time of the wax patterns was not affected by the different coolant flow rates while cooling water reaches turbulence fully. The Reynolds number can be calculated by the following Equation (2) [26].

(2) $$Re=\frac{4Q\rho }{\pi D\eta }$$

*Q* denotes the flow rate of the coolant;
*D* denotes the diameter of the cooling channel;
ρ denotes the density of the coolant;
η denotes the viscosity of the coolant.

**Figure 17:**
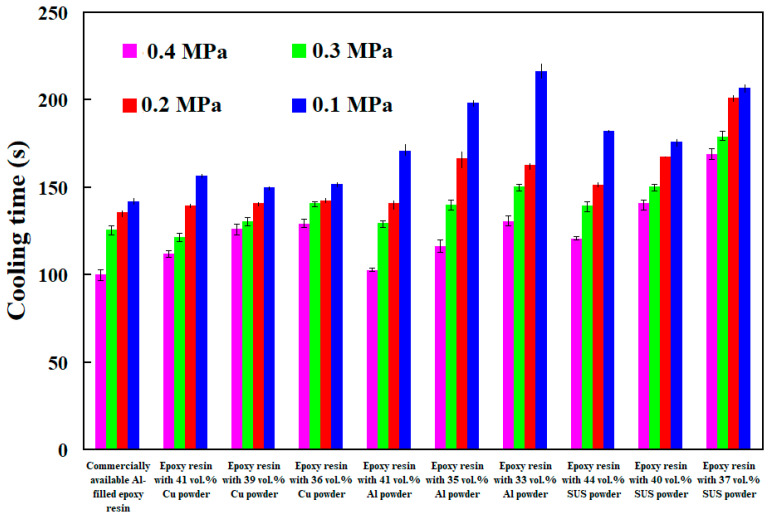
Cooling time of injection molded parts fabricated by ten sets of injection molds cooled with different compressed gas pressure.

**Figure 18:**
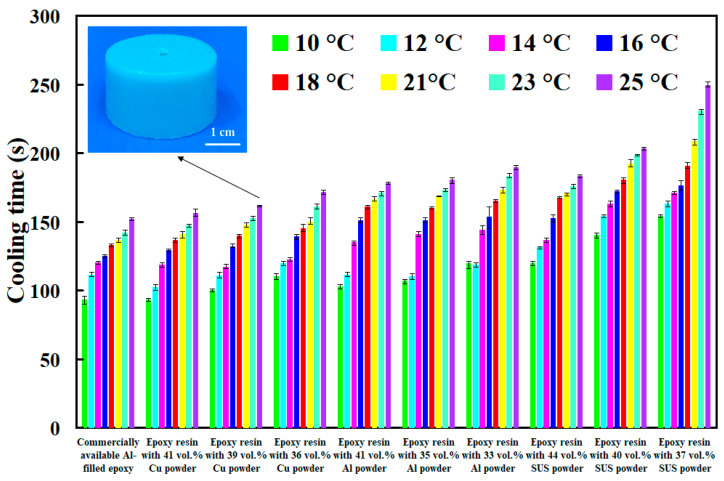
Cooling time of injection molded parts fabricated by ten sets of injection molds cooled with different cold air temperatures.

Figure 17 shows cooling time of injection molded parts fabricated by ten sets of injection molds cooled with different compressed gas pressure. The results showed that the cooling time of injection molded parts become shorter when compressed gas pressure is higher. The main reason is that better cooling effect derived from faster gas flowing caused by high pressure in the mold. Figure 18 shows cooling time of injection molded parts fabricated by ten sets of injection molds cooled with different cold air temperatures. It is interesting that two results were found. One is that the cooling time of injection molded parts become shorter when cold air temperature is lower. The other is that the cooling performance for cooling water is better than cold air under the same temperature.

**Figure 19:**
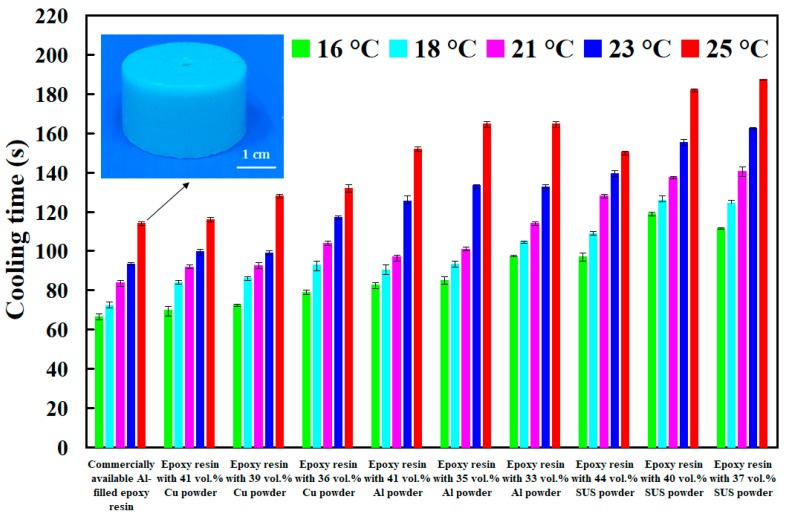
Cooling time of injection molded parts fabricated by ten sets of injection molds cooled with different temperature of cooling water with ultrafine bubbles.

The viscosity of water and air is about 0.8 m Pa-s and 18 μ Pa-s, respectively. The viscosity of water is about 44.5 times that of air. Therefore, the gas is mixed into the water to form cooling water with ultrafine bubbles is efficient to improve cooling efficiency and shorten the cooling time of injection molded parts. Figure 19 shows the cooling time of injection molded parts fabricated by ten sets of injection molds cooled with different temperatures of cooling water with ultrafine bubbles. As can be seen, the cooling water with ultrafine bubbles is the best candidate of cooling medium. Cooling time of the injection molded parts fabricated by commercially available Al-filled epoxy resin cooled with cooling water with ultrafine bubbles of 16 °C, 18 °C, 21 °C, 23 °C and 25 °C are 66 s, 72 s, 84 s, 93 s, and 114 s, respectively. However, cooling time of the injection molded parts fabricated by commercially available Al-filled epoxy resin cooled with conventional cooling water of 16 °C, 18 °C, 21 °C, 23 °C and 25 °C are 79 s, 85 s, 92 s, 110 s, 24 s, respectively. Cooling efficiency of the injection molded parts can be increased by approximately 15.6%, 14.6%, 8.4%, 15.4% and 8%, respectively. Thus, average cooling efficiency about 12.4% can be further increased compared with commonly used cooling water.

**Figure 20:**
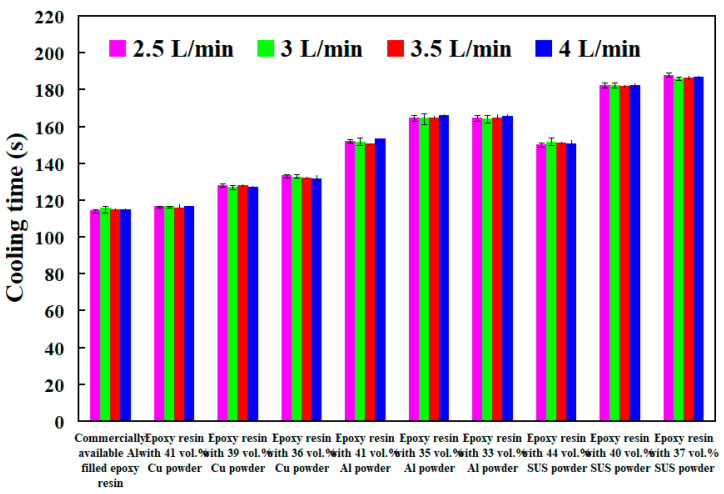
Cooling time of injection molded parts fabricated by ten sets of injection molds cooled with different flow rates of cooling water with ultrafine bubbles.

**Figure 21:**
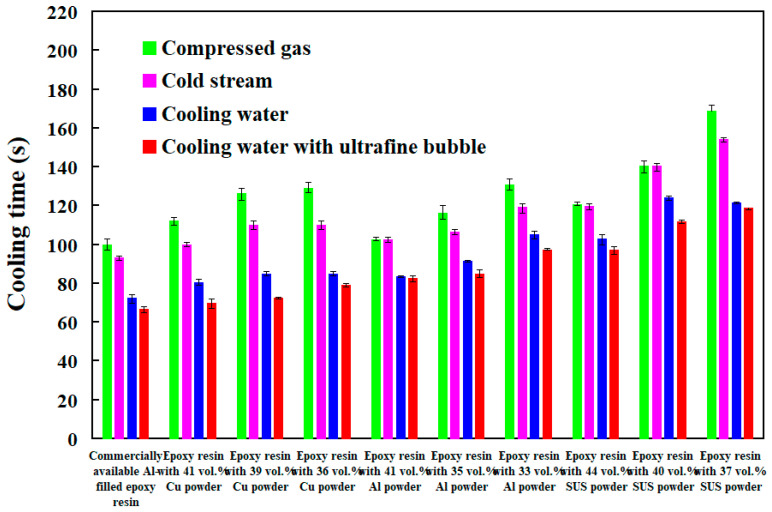
Shortest cooling time of injection molded parts fabricated by ten sets of injection molds cooled with different four different cooling media.

**Figure 22:**
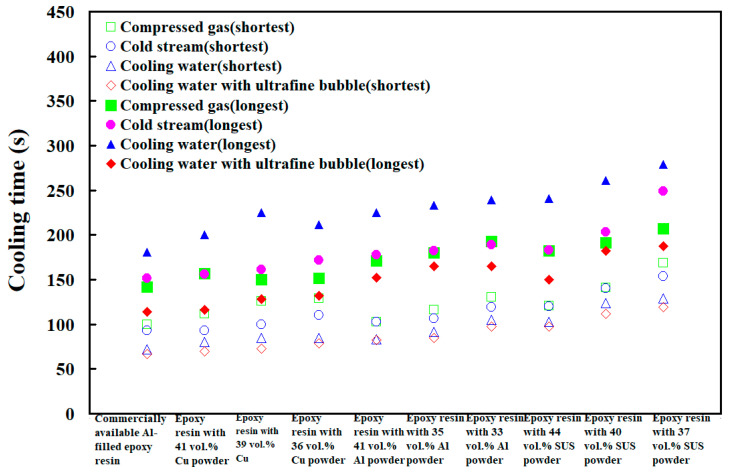
The longest and shortest cooling time of injection molded parts fabricated by ten sets of injection molds cooled with different four different cooling media.

To investigate cooling efficiency of cooling water with ultrafine bubbles at a temperature of 25 °C, four different coolant flow rates of cooling water with ultrafine bubbles are carried out. Figure 20 shows the cooling time of injection molded parts fabricated by ten sets of injection molds cooled with different flow rates of cooling water with ultrafine bubbles. The Reynolds number for four different coolant flow rates is about 4927, 5913, 6897, and 7883, respectively. It should be noted that the cooling time of the injection molded parts was not affected by the different coolant flow rates while cooling water reaches turbulence completely. Figure 21 shows the shortest cooling time of injection molded parts fabricated by ten sets of injection molds cooled with different four different cooling media. As can be seen, the cooling water with ultrafine bubbles is the best candidate of the cooling medium. It should be noted that the cooling performance will decrease significantly when the diameter of the cooling channel is less than 1.5 mm. According to practical experience, the time to cool the air is significantly lower than that of the water. Thus, the cooling medium can be replaced with cold air when the diameter of the cooling channel is less than 1.5 mm, since the cooling performance of cold air is acceptable. In addition, the mold material has a significant effect on the cooling time of the injection molded part since cooling time range of different mold materials is 99 s since the maximum and minimum cooling time is 279 s and 180 s, respectively. However, the cooling time range for different cooling media is only 92 s since the maximum and minimum cooling time is 279 s and 187 s, respectively. Figure 22 shows the longest and shortest cooling time of injection molded parts fabricated by ten sets of injection molds cooled with different four different cooling media. The total production cost of an injection mold fabricated with commercially available Al-filled epoxy resin is new Taiwan dollar (NTD) 3,046, while the total production cost of a injection mold fabricated by epoxy resin filled with 41 vol.% Al powder is only NTD 3999. Based on total production cost of injection mold and cooling efficiency, epoxy resin filled with 41 vol.% Al powder is the optimal formula for making an injection mold since saving in the total production cost about 24% is obtained compared to injection mold made with commercially available materials.

According to the results described above, the findings of this study are very practical and provide the greatest application potential in the precision mold industry, especially in the mold design stage. In this study, the internal surface of the CCC fabricated by additive manufacturing technology possesses high surface roughness compared with conventional cooling channel made by computer-numerical control machining [27]. The service life of the mold will be affected significantly due to stress concentration. The main reason is that the mold is repeatedly heated [28] and cooled during the wax injection process. Ongoing research is focused on improving the surface roughness of the CCC by abrasive blasting, abrasive flow machining [29], electrochemical polishing, chemical polishing, laser polishing, or ultrasonic cavitation abrasive finishing. In various industries, hydrogen [30,31] is widely applied as a high-performance gaseous cooling medium since its thermal conductivity is higher than all other gases. In addition, helium gas [32] or hydrogen [33] can also be applied as cooling media in gas-cooled nuclear reactors since its low tendency to absorb neutrons. Sulfur hexafluoride [34,35] is also widely applied to cool some high-voltage power systems, such as circuit breakers, transformer, or switches. These issues are currently being investigated and the results will be presented in a later study.

## 4. Conclusions

Generally, the cost-effectiveness depends on the cycle time of the injection molded products significantly in the mass production process of new products. The MAM method is frequently applied to speed up manufacturing for the production of injection mold with CCC. However, the major issue is expensive spending and longtime taking in the mold making process. The objective of this study is to study the cooling performance of epoxy-based injection molds fabricated by different mold materials using different coolant media. Based on the experimental results obtained from this work, the contributions and findings of this study can be summarized as follows:

1. A low-cost recipe that can produce cooling efficiency better than that made of commercial materials was demonstrated. The epoxy resin with 41 vol.% Al powder seems to be the optimum recipe for making injection mold with high cooling efficiency.
2. The cooling time of the injection molded parts is extremely sensitive to both injection mold material and the cooling medium. Cooling water with ultrafine bubbles is the best candidate of the cooling medium. Average cooling efficiency approximately 12.4% can be further increased compared with commonly used cooling water.
3. Mold material has a significant effect on the cooling time of the injection molded part, which has a greater influence on the cooling efficiency than the cooling medium since cooling time range of different mold materials is 99 s while the cooling time range for different cooling media is only 92 s. In addition, The cooling medium can be replaced with cold air while the diameter of the cooling channel is less than 1.5 mm and the coolant medium is water.

## Data Availability

Not applicable.

## Nomenclature

Acronyms	
CCC	Conformal cooling channel
Al	Aluminum
AM	Additive manufacturing
RT	Rapid tooling
CAD	Computer-aided design
MAM	Metal additive manufacturing
Cu	Copper
FE-SEM	Field-emission scanning electron microscopy
EDS	Energy-dispersive X-ray spectroscopy
XRD	X-ray diffractometer
SUS	Stainless steel
PVB	Polyvinyl butyral
NTD	New Taiwan dollar
List of symbols	
*T_w_*	Mold cavity surface temperature
*s*	Part thickness
*T_m_*	Melt temperature
*T_e_*	Average ejection temperature
*Q*	Flow rate of the coolant
*D*	Diameter of the cooling channel
Greek symbols	
*α*	Thermal diffusivity
ρ	Density of the coolant
η	Viscosity of the coolant
